# Machine Learning as a Tool for the Development of Sign Recognition Systems: A Review

**DOI:** 10.3390/jimaging12090445

**Published:** 2026-09-15

**Authors:** Juan E. Mora-Zarate, Claudia L. Garzón-Castro

**Affiliations:** Research Group CAPSAB, Engineering Faculty, Universidad de La Sabana, Chia 250001, Colombia

**Keywords:** sign language, machine learning, anatomical landmarks, sign recognition system

## Abstract

Hearing loss represents a global challenge, affecting the ability of the deaf community to communicate, study, and work. Currently, the World Health Organization reports that more than 430 million people live with disabling hearing loss. Among rehabilitation alternatives, sign language is one of the most widely adopted; however, effective communication between deaf individuals and the hearing population remains a barrier. To help bridge this communication gap, the development of technological tools has been promoted, particularly Sign Recognition Systems (SRSs). The purpose of this review is to provide an integrative analysis of the current state of SRS research motivated by sign language teaching and learning applications, identifying dominant methodological approaches across the full pipeline—from data capture and feature extraction to classification and technological deployment—and highlighting the persistent gap between high experimental performance and real-world accessibility. This literature review analyzes 55 articles, selected from an initial pool of 161 records identified in journals indexed in the Scopus database between 2020 and 2025. The analysis revealed that these systems are generally structured in two stages: feature extraction and classification. Finally, despite the high performance achieved by SRS, a gap remains in transferring these systems into technologies that can be effectively used by end users.

## 1. Introduction

Deafness and hearing loss affect people’s ability to communicate, study, and work [1]. Worldwide, more than 430 million individuals experience disabling hearing loss (greater than 35 dB), a figure that could increase to 700 million by 2050 due to conditions such as exposure to high-intensity sounds and the use of ototoxic medications [2]. However, middle-income countries still lack the infrastructure required for the prevention, treatment, and rehabilitation of hearing problems, even though they account for 80% of the population with hearing disabilities [1].

Current rehabilitation guidelines include auditory technologies (such as cochlear implants or hearing aids), therapies aimed at developing language skills (phoniatrics or speech therapy), and sensory substitution strategies (lip reading or sign language) [2]. Nevertheless, the first two options depend on the support of national health systems or on an individual’s ability to independently access specialized care. For this reason, sign language has become one of the most widely adopted rehabilitation alternatives within the deaf community, as it is commonly taught in institutions dedicated to the education of people with hearing impairments.

**Figure 1:**
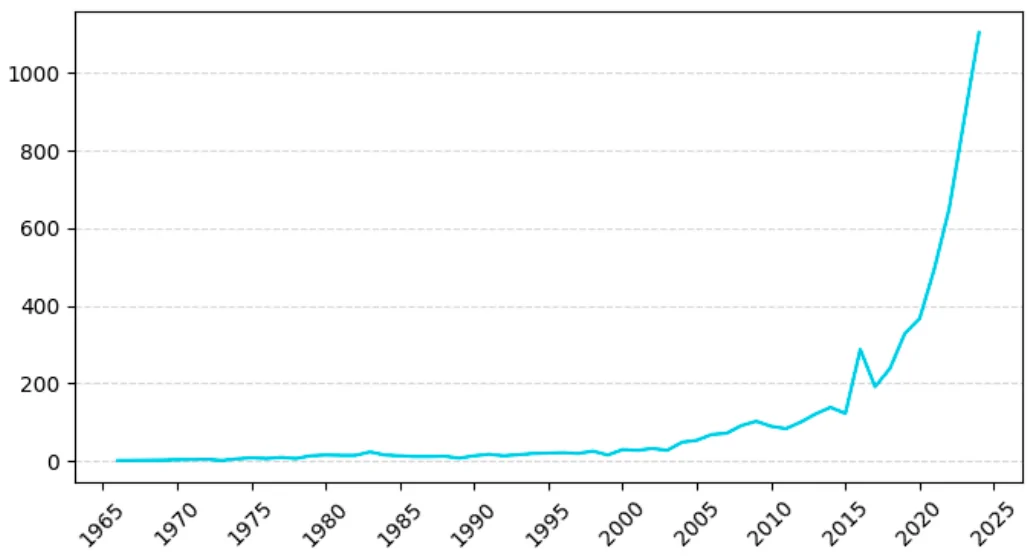
Articles published in SCOPUS under the search equation: “sign language” AND (“teaching” OR “learning”).

On the other hand, even when individuals with hearing disabilities are proficient in sign language, this does not guarantee effective communication with hearing people. Consequently, over the years, both academia and industry have increased efforts toward the development of technological tools focused on sign language learning for anyone interested in acquiring it (see Figure 1).

**Figure 2:**
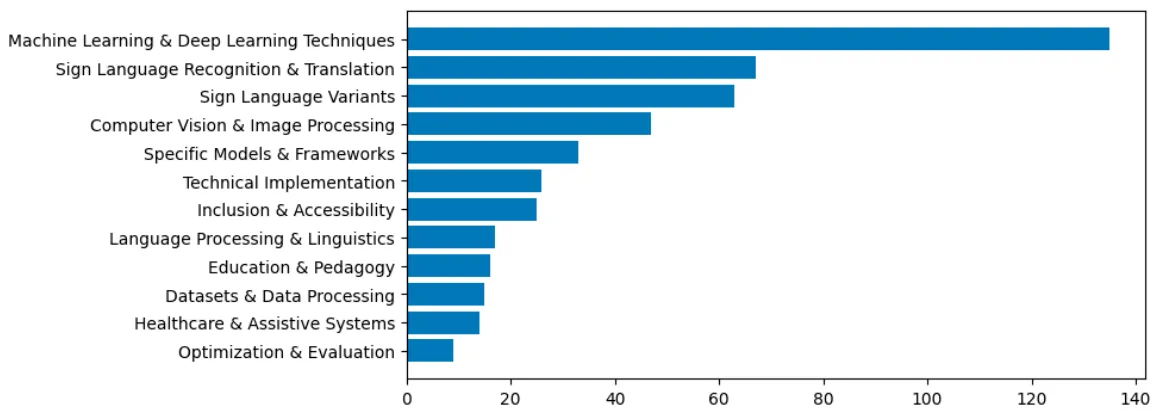
Frequency chart grouping keywords by common topics, search equation: “sign language” AND (“teaching” OR “learning”), limited to the fields of engineering and computer science.

A frequency analysis of technologies developed in the last five years (see Figure 2) shows that the predominant trend is the construction of Sign Recognition Systems (SRSs) through Machine Learning (ML) techniques.

This document presents a literature review of SRS based on machine learning techniques and their integration into technologies. The structure of the document is as follows: (1) definition of SRS types, (2) applications of SRS, (3) discussion, and (4) conclusions. All articles were retrieved from SCOPUS using the search equation: (“hard of hearing” OR deaf) AND “sign language” AND (“teaching” OR “learning”), limited to English and Spanish publications, Open Access, within the fields of engineering and computer science, and published between 2020 and 2025. This query yielded a total of 161 articles.

The selection of articles followed explicit inclusion and exclusion criteria, summarized in Table 1, to ensure the relevance and methodological consistency of the reviewed literature.

**Figure 3:**
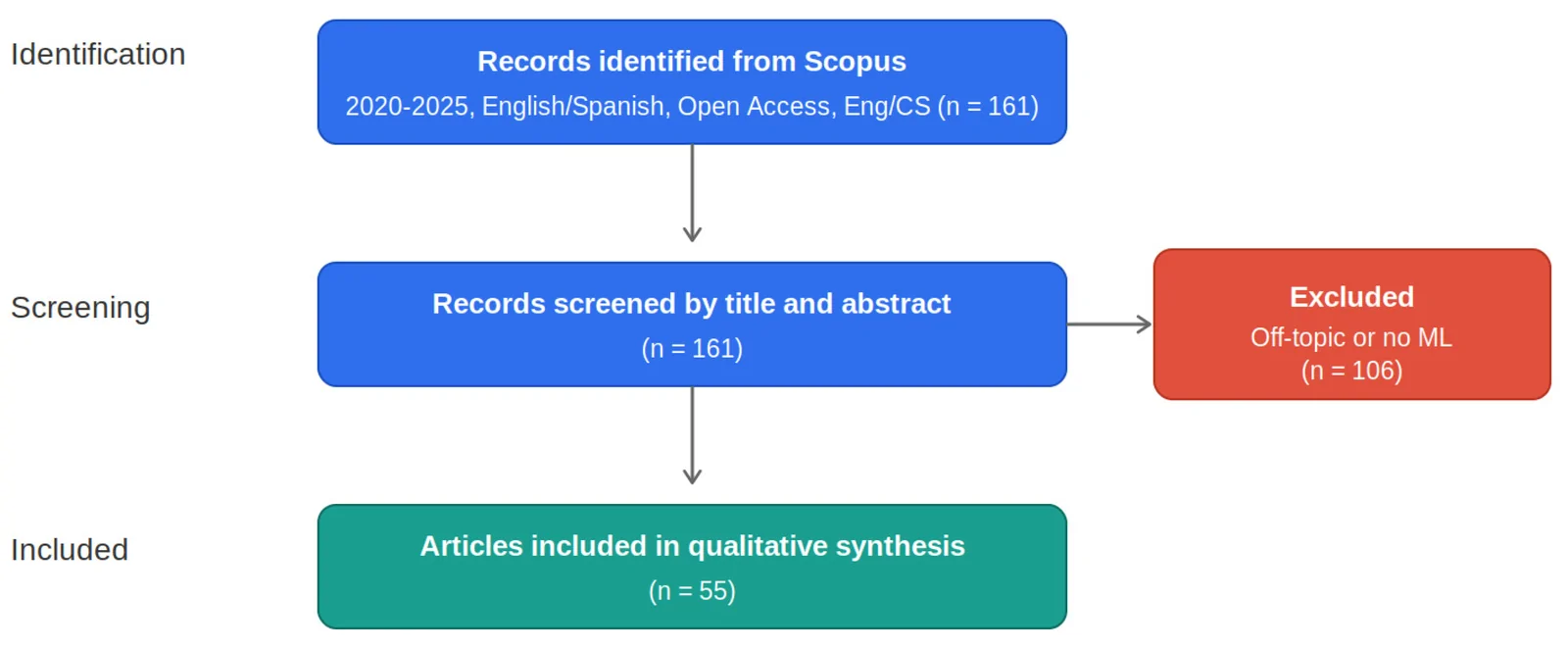
PRISMA flow diagram of the literature identification, screening, and inclusion process.

Figure 3 presents a PRISMA-style flow diagram of the literature selection process. Of the 161 records identified through the Scopus search equation, 106 were excluded during title and abstract screening for being off-topic or for not employing machine learning methods, yielding a final set of 55 articles used for the detailed technical synthesis presented in this review. The bibliometric analyses presented in Section 1 (publication trends, keyword co-occurrence, and geographic distribution) were conducted over the full corpus of 161 identified records, as these characterize general research trends rather than requiring the level of detail assessed during full screening; the 55 screened articles constitute the basis for the technical taxonomy, comparative analysis, and performance evaluation presented in Section 2, Section 3, Section 4 and Section 5. This review is guided by the following research questions: (RQ1) What feature extraction techniques are most commonly used in machine learning-based Sign Recognition Systems motivated by sign language teaching and learning applications, and what are their relative advantages and limitations? (RQ2) What classification architectures are predominantly applied to static and dynamic sign recognition in this research population, and how do their performance and computational requirements compare? (RQ3) To what extent has this teaching/learning-motivated SRS research been integrated into user-accessible technologies, and what factors constrain this transition from experimental performance to real-world deployment? (RQ4) What evaluation metrics and methodological practices are currently used to assess performance in this body of research, and what gaps exist in current reporting standards?

**Figure 4:**
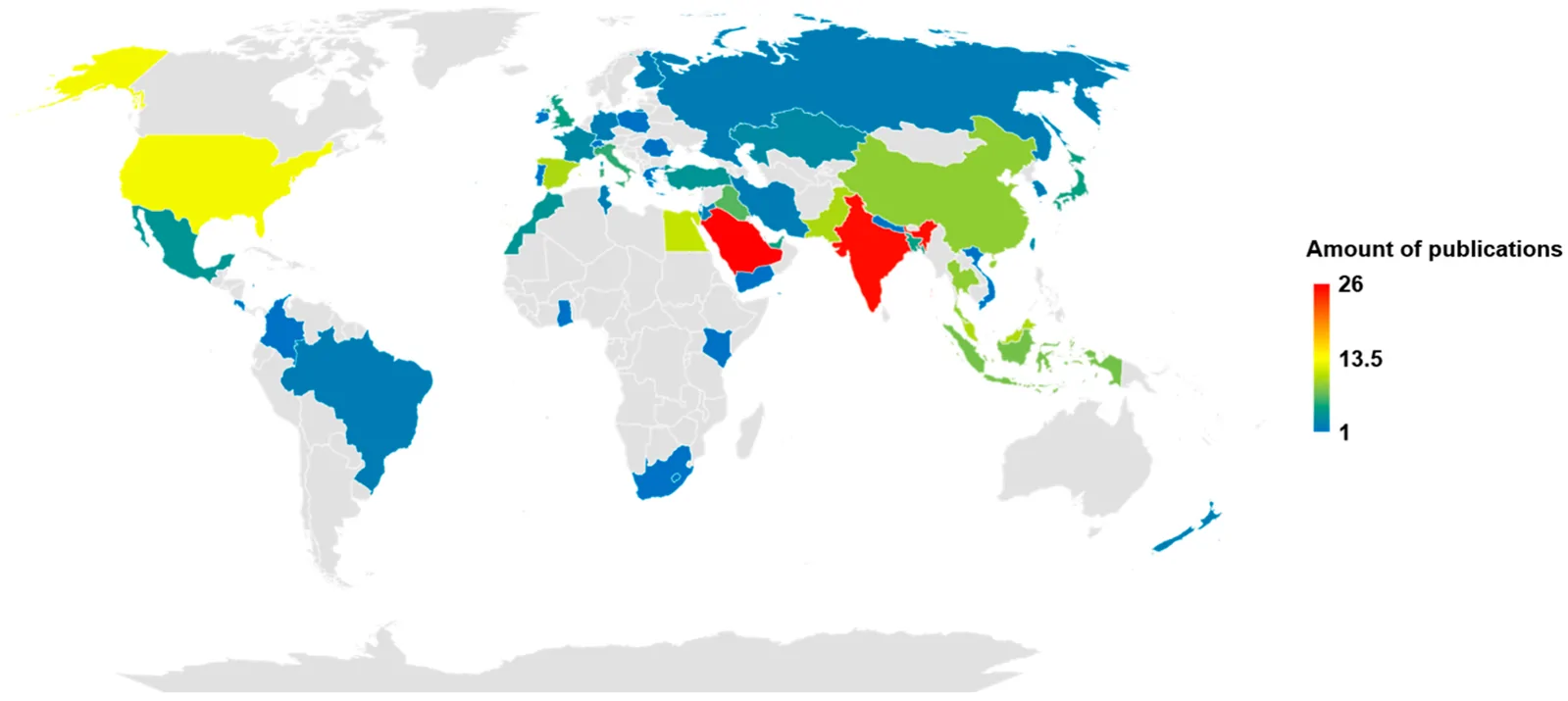
Number of publications by country based on the SCOPUS search equation: (“hard of hearing” OR deaf) AND “sign language” AND (“teaching” OR “learning”).

Similarly, Figure 4 shows the geographical distribution of the studies found under this search equation, revealing a significant concentration of research in Asian and Middle Eastern countries, with Saudi Arabia and India leading scientific production in this area. In contrast, nations in Europe, Oceania, and Latin America show lower participation. This uneven distribution highlights a gap in knowledge generation regarding sign language education, particularly in Latin American and African countries.

Although several recent reviews have addressed advances in Sign Language Recognition (SLR) and Sign Language Processing (SLP), most studies have focused on specific languages, technologies, or application domains. Ref. [3] emphasized Turkish Sign Language and multimodal deep-learning architectures, while [4] focused on datasets and machine learning approaches used in SLR studies. Likewise, ref. [5] reviewed recognition, translation, production, and datasets, whereas [6] analyzed interpretation systems, translation methods, and mobile applications. Additionally, Berrezueta-Guzman, Santiago [7] explored Virtual Reality and Artificial Intelligence applications for sign language education. Despite these contributions, previous reviews mainly address isolated components of the SRS pipeline or specific technological contexts.

In contrast, this review provides an integrative analysis of Sign Recognition System research motivated by sign language teaching and learning applications, covering the complete workflow from data capture and preprocessing to feature extraction, classification models, and technological implementation. Beyond reporting recognition performance, this study highlights the persistent gap between high experimental accuracies and the effective deployment of accessible technologies for real-world communication scenarios. By synthesizing 55 studies, selected from an initial pool of 161 identified records, published between 2020 and 2025, this review offers a broader perspective on current trends, methodological limitations, and future directions needed to support the transition from laboratory-based models to practical assistive technologies for the deaf community.

## 2. Sign Recognition Systems

SRSs are composed of two stages: one responsible for capturing information from the user performing a sign, and another that receives this information to feed a predictive model whose function is to output the sign executed by the user [8,9]. Additionally, prior to continuous information capture, a dataset must be built to train the predictive model [10,11]. Table 2 presents the studies reported in the literature that involve the development of an SRS, highlighting the use of deep learning methodologies combined with computer vision.

### 2.1. Feature Extraction

**Figure 5:**
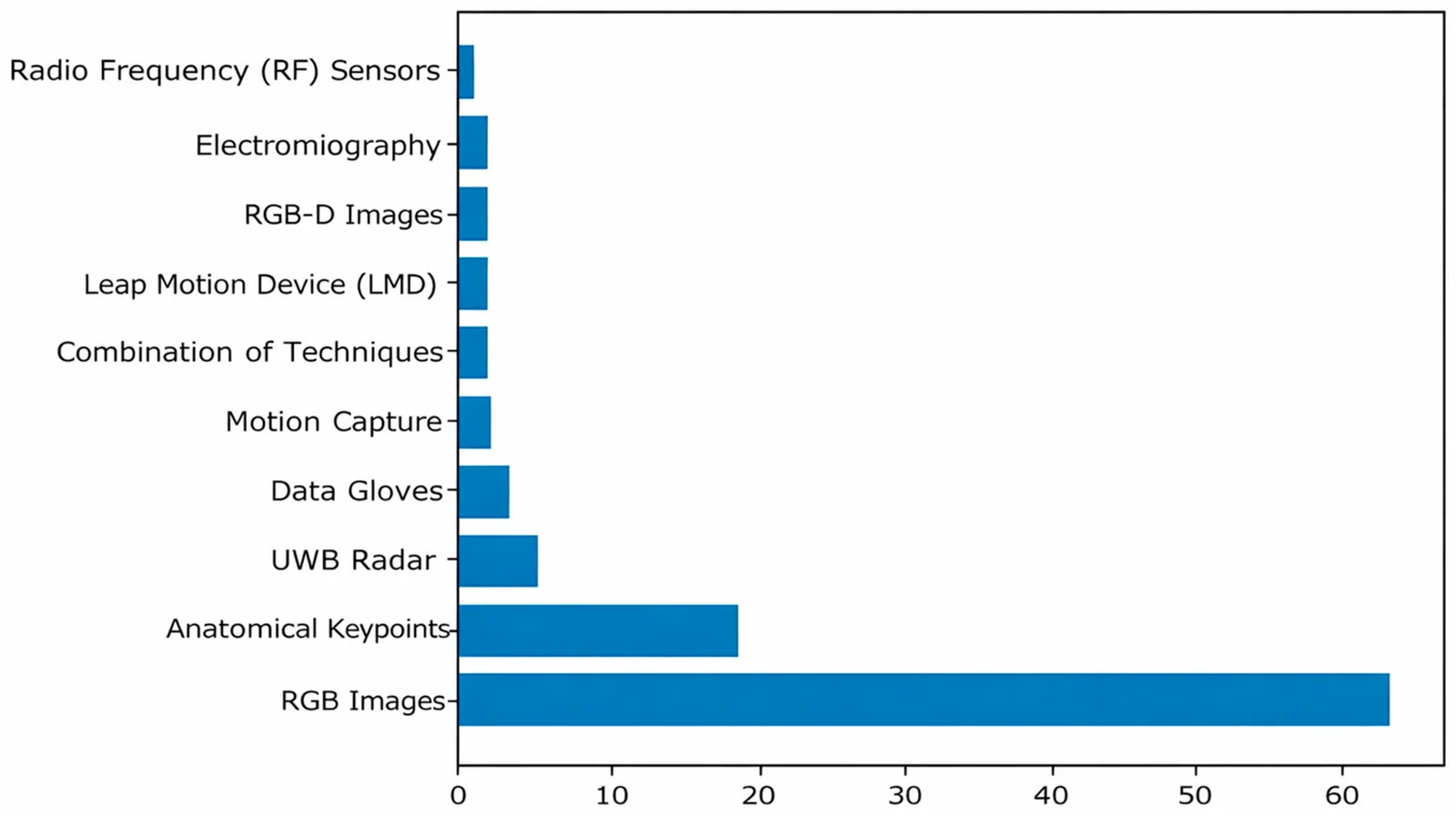
Frequency chart of feature extraction techniques reported in articles published between 2020 and 2025 in the SCOPUS database.

In sign recognition systems, the feature extraction process is essential to transform human motion into data that can be analyzed by predictive models. According to the search results, out of the 161 papers identified, 102 explicitly described their feature extraction procedures. The most frequently used techniques (see Figure 5) were anatomical landmark prediction from images in the red–green–blue color space (RGB) and the use of raw images without preprocessing. Each of these 102 articles was classified according to its primary input modality—with RGB images and anatomical landmarks as the two dominant categories, alongside less frequent categories such as data gloves, RF/radar sensors, EMG, and RGB-D imaging. As shown in Figure 4, the two most frequently used techniques together substantially outnumber the remaining, sensor-based categories.

Techniques based on RGB image capture and anatomical landmark extraction account for 79% of the selected articles. In both cases, the primary acquisition devices are stereo cameras, which generate RGB-format images.

#### 2.1.1. RGB Images

When RGB images are used as input to a predictive model for sign recognition, the construction of a diverse dataset is required. To achieve this, images are captured under different backgrounds, lighting conditions, shadows, camera inclinations, and participant genders [12,15,16]. However, this variability is often insufficient; therefore, data augmentation techniques are commonly applied, such as image rotation, flipping, color variation, and modifications in brightness, contrast, saturation, and hue [22,28].

Once the dataset is available, although images already represent the motion performed by the user, these representations are often not descriptive enough. Consequently, they are post-processed using techniques such as segmentation and color-space transfor-mations (e.g., HSV, YCrCb) [29,32,36].

Moreover, in modern deep learning approaches, features are extracted using Convolutional Neural Networks (CNNs) [20,37,38], which aim to learn filters that, through convolution, capture relevant spatial patterns. Pretrained architectures are frequently adopted for this purpose, including ResNet50 [39], EfficientNetB3 [37], Inception V3 [20], and VGG16 [38].

Despite their widespread use, RGB-based approaches remain highly sensitive to environmental variability. Even with data augmentation, models trained on RGB images often generalize poorly to lighting conditions, backgrounds, or camera angles not represented in the training data, and the high dimensionality of raw images increases both the volume of data and the computational cost required for training.

#### 2.1.2. Anatomical Landmarks

This approach refers to the use of models such as MediaPipe [13,24] for detecting key points of the human body. These models estimate 3D anatomical coordinates of body parts such as hands, face, and torso. A key advantage of this method is the ability to track landmark positions over time, which is particularly useful for dynamic signs involving movement [40,41].

Additionally, the information fed into the predictive model becomes more descriptive because it provides both temporal and spatial cues. Finally, this method reduces the dimensionality of the input compared with RGB-based approaches, since only relevant landmarks can be selected [42] or geometric properties can be computed [17].

Nonetheless, this approach depends entirely on the reliability of the landmark detector. Occlusions, unusual hand orientations, or rapid motion frequently cause missed or misplaced landmark detections, which propagate directly as errors into the downstream classifier without any correction mechanism.

#### 2.1.3. Other Extraction Techniques

Beyond RGB images and anatomical landmarks, other feature extraction strategies have been explored. Although less frequent in the literature, they provide advantages in specific contexts.

One example is data gloves, which employ flex sensors [43] or inertial measurement units (IMUs) [18,44] to directly capture finger joint angles and hand orientation. This approach produces highly reliable features because it is not affected by environmental conditions. However, it presents challenges such as cost, the need for users to wear the device, and the inability to capture facial or torso movements.

Other contactless techniques include ultra-wideband (UWB) radar [25] and radiofrequency (RF) sensors [45], which detect body movements through electromagnetic reflections. Nevertheless, these technologies offer limited spatial resolution, restricting their capability to interpret complex gestures. Similarly, motion capture systems [46,47] provide precise 3D body data, but their high-cost limits everyday applicability.

Compact sensors such as the Leap Motion Device (LMD) [48] can detect finger position and orientation in 3D using infrared cameras, offering good accuracy without physical contact, albeit with a limited field of view. Electromyography (EMG) [49] measures muscle electrical activity during gestures, but it requires calibration and may produce noisy data. Finally, RGB-D images [50], which combine color and depth, improve segmentation and body tracking, although performance can be affected by the distance between the user and the camera. These complementary technologies offer valuable alternatives depending on application requirements.

Taken together, these alternative techniques illustrate a recurring trade-off in SRS feature extraction: approaches that improve reliability or reduce environmental sensitivity (data gloves, EMG) typically do so at the cost of user convenience, cost, or capture completeness (e.g., the inability to record facial expressions), while contactless alternatives (RF, LMD) trade this convenience for reduced spatial resolution.

#### 2.1.4. Comparative Analysis of Feature Extraction Techniques

The choice of feature extraction strategy involves fundamental trade-offs between representational richness, environmental robustness, hardware requirements, and user acceptance that directly condition the viability of an SRS in real-world deployment. RGB image-based approaches offer the lowest hardware barrier but are highly sensitive to environmental conditions and carry a large amount of irrelevant information, increasing the dimensionality of the learning problem. Anatomical landmark extraction reduces the input to a compact set of coordinates that are largely invariant to background and lighting conditions, decreasing training data requirements and inference time, at the cost of dependency on the reliability of the landmark detector itself. Data gloves and IMU-based systems produce the most reliable and noise-resistant features but require users to wear specialized hardware and cannot capture facial expressions or torso movements. RF and radar-based sensors offer contactless operation but are limited in spatial resolution; EMG-based approaches capture rich neuromuscular information but require calibration and are sensitive to fatigue and sweat; RGB-D cameras improve segmentation and depth estimation but are more expensive and sensitive to ambient infrared light. In summary, for applications prioritizing accessibility and low deployment cost, landmark-based approaches currently represent the best balance between performance and practicality, while contact-based and specialized sensor approaches are better suited to controlled research or clinical environments where reliability outweighs convenience.

### 2.2. Classification Techniques

**Figure 6:**
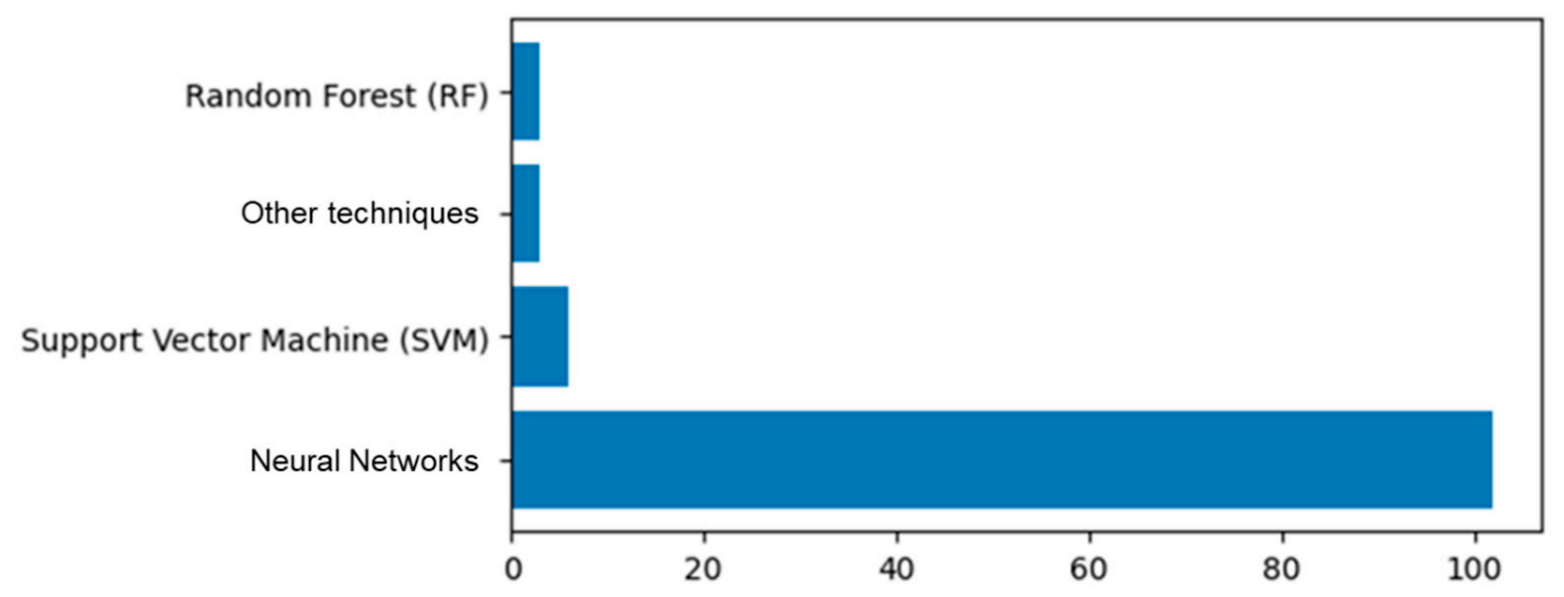
Frequency of the most used predictive methods according to the search results.

Classification techniques define the structure of the predictive model. The model input corresponds to features extracted using the methods described previously, while the output is the predicted sign associated with those features. The frequency of methods identified in the search is shown in Figure 6, where neural networks dominate, appearing in 89% of the articles. Each reviewed article was classified according to its primary predictive model architecture, grouped into four categories: deep learning (DL) methods—including CNN, LSTM, GRU, and Transformer architectures—Support Vector Machines (SVM), Random Forests (RF), and other traditional or hybrid methods. This dominance of DL methods reflects the broader shift toward deep learning observed across computer vision research during the 2020–2025 period. SVM and RF classifiers, while considerably less frequent, remain present primarily in studies using highly descriptive input features, such as anatomical landmarks or data glove sensor readings, where the added representational capacity of deep networks offers diminishing returns.

#### 2.2.1. Neural Networks

Neural networks are widely used in SRS due to their ability to learn complex patterns. For static signs, which consist of fixed configurations of hands and fingers, networks preceded by convolutional processes are common [19,51], particularly when working with RGB images [50]. However, convolution may be omitted when the extraction method already provides highly descriptive features, such as with data gloves [43].

It is also common in static sign recognition to use pretrained object detection models such as YOLOvX [52,53], which have demonstrated strong capabilities in visual recognition.

Dynamic signs, involving motion sequences over time, require architectures capable of modeling temporal dependencies. Recurrent Neural Networks (RNNs), especially Long Short-Term Memory (LSTM) [31], Gated Recurrent Units (GRU) [23], Bidirectional LSTM (Bi-LSTM) [54], and Transformers [27], are frequently adopted.

Beyond recurrent models, Transformer-based architectures have become increasingly prominent in SRS research. The self-attention mechanism allows Transformers to model long-range temporal dependencies without the sequential constraints of RNNs, making them well-suited for continuous sign recognition. Transformers have been applied both as standalone classifiers [55] and in hybrid configurations that combine CNN-based spatial feature extractors with Transformer encoders for temporal modeling [23]. More recently, Vision Transformers (ViT) have been adapted for static and video-based sign recognition by treating image patches or frame sequences as token sequences, enabling the model to capture global spatial relationships that convolutional operations may miss; approaches such as video masked autoencoders with fine-tuning have demonstrated the viability of this paradigm for Arab Sign Language recognition from video [56]. However, ViT-based models generally require large training datasets to outperform CNNs, which is a constraint in sign language domains where annotated data is scarce. At the frontier of the field, the integration of large-scale foundation models—such as MediaPipe Holistic for multi-body landmark extraction and large language models (LLMs) for sign-to-text translation—has begun to appear in the literature [34]. Despite their promise, their computational cost and dependence on proprietary infrastructure currently limit their adoption in accessible, real-world assistive technologies.

A common limitation across these architectures is their dependence on large volumes of labeled training data, which remains scarce for most sign languages beyond ASL. Recurrent and Transformer-based models, while effective at modeling temporal dependencies, further increase computational cost and training time compared with static classifiers, which can constrain their deployment on resource-limited devices. The reviewed articles also generally did not report sufficient implementation-level detail—such as specific hyperparameters or layer configurations—to allow a systematic assessment of how architectural choices within the same classifier family affect performance.

#### 2.2.2. Other Classification Techniques

As alternatives to neural networks, traditional machine learning methods such as Support Vector Machines (SVM) [57] and Random Forests (RF) [55] have also been employed. Although their presence has decreased, their fast training time and low computational requirements remain advantageous for static sign classification.

However, their effectiveness strongly depends on the availability of well-defined features, such as those derived from anatomical landmarks [41] or data gloves [44].

Beyond their dependence on well-defined input features, SVM and Random Forest classifiers generally do not scale well to large sign vocabularies or high-dimensional raw inputs, which restricts their applicability to problems with a limited number of classes and compact, pre-engineered feature representations.

#### 2.2.3. Comparative Analysis of Classification Techniques

The selection of a classification architecture involves trade-offs between modeling capacity, computational cost, training data requirements, and interpretability. CNN-based architectures excel at extracting hierarchical spatial features and achieve near-perfect accuracy on static sign recognition with controlled datasets, but cannot model temporal dependencies natively. LSTM and GRU networks address this by processing sequences step by step, achieving linear computational complexity with respect to sequence length, but their sequential nature limits parallelization during training and increases cost for long sequences; their memory footprint at inference is generally moderate, as only the current hidden and cell states must be maintained. Transformer-based architectures compute self-attention across all time steps simultaneously, resulting in quadratic computational complexity with respect to sequence length; while this enables parallelized training and superior modeling of long-range dependencies, it also results in a larger inference-time memory footprint than LSTMs for equivalent sequence lengths, which can be prohibitive for mobile or embedded deployment without compression techniques such as pruning or quantization. Hybrid CNN-LSTM architectures—where a CNN extracts per-frame spatial features before an LSTM models temporal dependencies—offer a practical middle ground: the CNN component is computationally cheap and parallelizable per frame, while the LSTM adds only linear temporal complexity, resulting in a more favorable complexity-performance trade-off than pure Transformer-based pipelines for moderate-length sequences, at the cost of the global temporal context that self-attention provides. In practice, CNN-LSTM hybrids are generally preferable for resource-constrained, real-time applications, while Transformers are better suited to research contexts prioritizing maximum accuracy on longer, more complex sign sequences where computational resources are less constrained. Traditional classifiers (SVM, Random Forest) have largely been superseded by deep learning in raw performance but retain advantages in low-data regimes, interpretability, and computational simplicity when the feature extraction stage produces sufficiently descriptive representations. Overall, no single architecture dominates across all SRS scenarios: the choice should be driven by sign type (static vs. dynamic), available training data, target deployment platform, and acceptable inference latency.

## 3. Sign Language Datasets

The development and evaluation of SRS depend critically on the availability of annotated datasets, which define vocabulary size, recording conditions, number of signers, and capture modality. Table 3 summarizes the most frequently referenced datasets identified across the reviewed literature, characterized by sign language, modality, sign type, and scale.

Several observations emerge from this analysis. First, the majority of large-scale, publicly available datasets cover American Sign Language (ASL) or Arabic Sign Language (ArSL), reflecting the geographic concentration of research identified in Figure 3. Second, most datasets are captured under controlled laboratory conditions, which may limit the generalizability of models trained on them to real-world environments. A critical gap identified in the literature is the scarcity of datasets for sign languages of Latin American and African countries. With the exception of LSA64 for Argentinian Sign Language, these linguistic communities remain largely underrepresented, directly constraining the development of accessible SRS for these communities.

## 4. Use of Sign Recognition Systems

**Figure 7:**
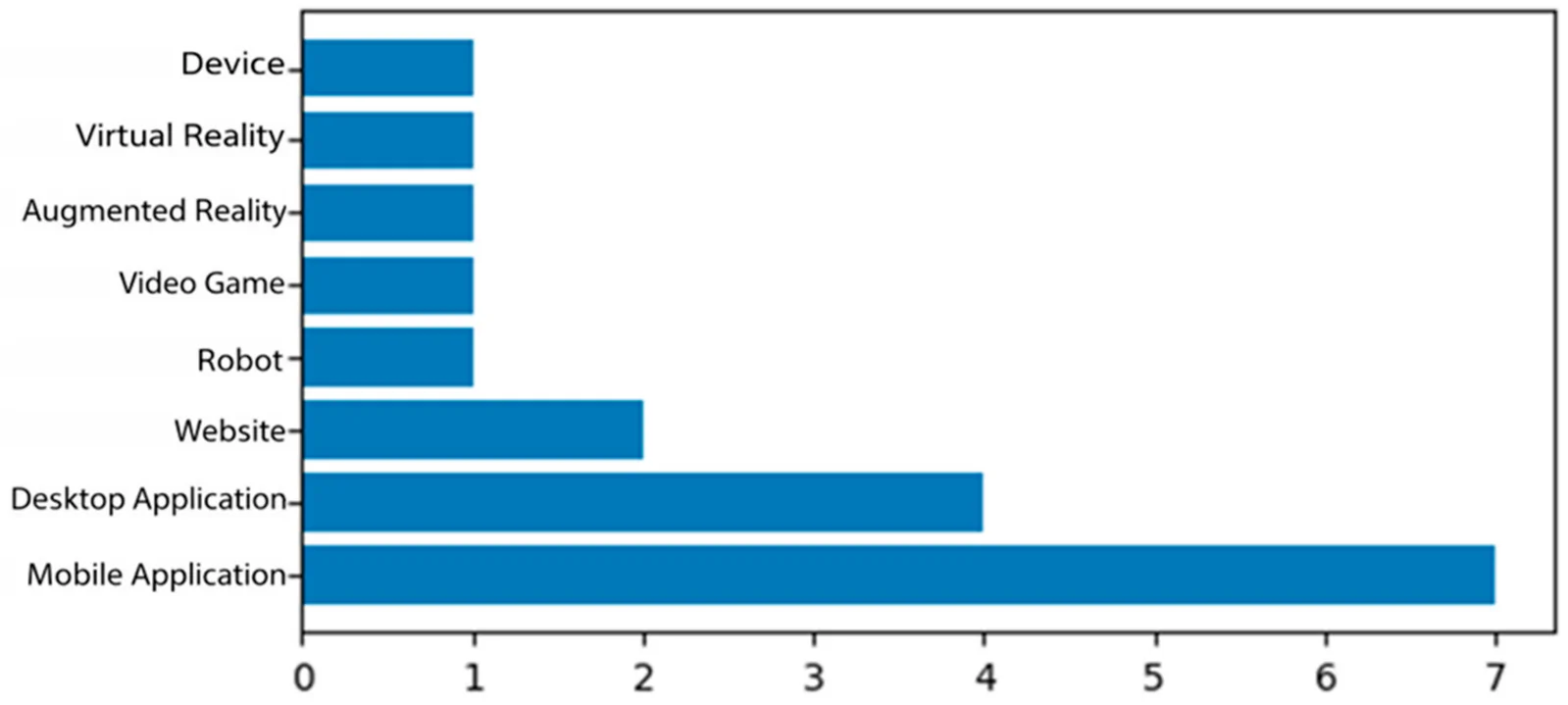
Frequency of technologies integrating Sign Recognition Systems.

Despite growing interest in SRS development, practical implementation in user-accessible technologies remains limited. Among the 161 identified articles, only 18 (11.2%), as shown in Figure 7, reported integration into interactive technologies. Platforms mainly included mobile applications [11,14,59], desktop applications [60,61], and web platforms [39,62]. Fewer implementations were found in robots, video games, augmented reality, virtual reality, and physical devices.

This distribution suggests a preference for software-only solutions, likely due to lower costs and entry barriers compared with hardware-dependent technologies.

## 5. Performance of Predictive Models

Beyond understanding what SRS are and where they are deployed, it is necessary to assess their reliability to determine the maturity of the field.

The most reported metric is accuracy, defined as the proportion of correct predictions over the total evaluated instances, i.e., the percentage of correctly predicted signs.

### 5.1. Performance in Dynamic Sign Recognition

Dynamic sign recognition involves sequential information processing challenges (Table 4). summarizes performance, showing that systems typically manage between 20 and 50 classes, with accuracies ranging from 85% to 98%.

### 5.2. Performance in Static Sign Recognition

Unlike dynamic signs, static signs are defined by specific body and hand configurations without temporal components, resembling conventional image classification problems. Table 5 shows that the number of classes is usually smaller, often limited to alphabets and numbers from 1 to 10.

Accuracy values are typically higher than in dynamic recognition, frequently between 95% and 99%, since only spatial patterns must be identified.

## 6. Future Research Directions

The analysis presented in Section 5 shows that CNN-based architectures achieve accuracies between 95% and 99% in static sign recognition, where the input corresponds to a fixed hand and body configuration without a temporal component. Recent work confirms this pattern: a two-stage CNN-BiLSTM training strategy applied to the ArASL2018 benchmark reached 99.50% accuracy without data augmentation, by separating representation learning from classifier optimization [65]. This result indicates that the methodological space for static sign recognition has reached a level of maturity in which further gains depend on training strategy refinement rather than on new feature extraction or classification paradigms.

In contrast, dynamic sign recognition, in which a sign is defined by a sequence of hand, face, and body configurations over time, remains an open problem, and recent literature shows a shift in research focus toward this category at both the word and phrase level. At the word level, a Transformer encoder trained on MediaPipe hand landmark sequences from the WLASL dataset, combined with a fusion-based reranking strategy using K-means clustering confidence scores, improved Top-5, Top-10, and Top-20 accuracy relative to a Transformer-only baseline, while Top-1 accuracy remained unchanged, which indicates that the main source of error lies in distinguishing between visually similar signs rather than in temporal modeling [58]. Spatiotemporal fusion architectures address dynamic word-level recognition through a different approach: a dual-path Convolutional Neural Network-Long Short-Term Memory-Transformer framework, applied to an Indian Sign Language emergency gesture dataset, combined a Transformer branch for long-range temporal dependencies with an LSTM branch for short-term motion continuity, reaching an accuracy of 97.59% under a participant-disjoint evaluation protocol [66]. A related direction addresses the latency constraint associated with dynamic recognition: a real-time spatio-temporal adaptive motion pattern framework processes frames only when hand or body motion is detected, reducing the number of frames analyzed by a temporal memory Transformer and producing predictions before a gesture is completed [67].

Video-based approaches that avoid landmark extraction have also been reported. A Convolutional Grid Long Short-Term Memory network, whose hyperparameters were tuned through a nature-inspired optimization algorithm, combined hand action-unit detection with facial segmentation to recognize signs directly from video frames, reporting 96.707% accuracy under k-fold cross-validation [56]. This approach indicates that dynamic sign recognition is increasingly incorporating facial information together with hand motion, rather than treating sign language as a hand-only gesture recognition problem.

The combination of non-manual features—facial expression, lip movement, and torso orientation—with manual (hand) features is one of the challenges associated with this shift toward dynamic recognition. A comparison of four multimodal architectures for gesture recognition, each processing manual and non-manual (lip movement) features separately before merging them, found that early feature fusion combined with parallel LSTM processing reached the highest accuracy (0.99), while attention- and Transformer-based fusion reached 0.97 [68]. Because this comparison relied on video input alone, without depth cameras or inertial sensors, it indicates that further gains in this category depend on the fusion strategy applied to heterogeneous modalities rather than on additional sensor hardware.

At the phrase level, sign language translation extends recognition beyond isolated signs to continuous sequences that are mapped to sentences in a spoken or written language, a task constrained by the limited availability of gloss annotations. A cross-modality self-supervised learning method with sigmoid self-attention weighting was proposed to align sign language video features with question (dialogue) text instead of gloss labels, reporting that question-based supervision can match or exceed gloss-based supervision on the CSL-Daily-QA and PHOENIX-2014T-QA datasets [69]. This result indicates that reducing dependence on gloss annotation is a relevant direction for phrase-based recognition, given that gloss labeling requires manual annotation by sign language experts.

Taken together, these studies indicate that the research trend in SRS is moving from static, image-based recognition toward dynamic recognition at the word and phrase level, consistent with the fact that sign language communication is continuous rather than composed of isolated static configurations. This transition introduces feature extraction and classification requirements that are not present in static recognition: dynamic signs require the joint modeling of hand shape, motion trajectory, facial expression, and, in some cases, torso orientation, over time, while remaining compatible with real-time processing constraints. Future work in this field should prioritize architectures and datasets that integrate hand, face, and torso information within a single temporal model, together with reduced dependence on gloss-level annotation, to support the transition from isolated dynamic sign recognition toward continuous, phrase-level sign language translation.

## 7. Conclusions

Research in SRS has reached significant technical maturity, consolidating deep learning as the dominant paradigm. There is a clear preference for non-invasive computer vision approaches, particularly RGB image analysis and anatomical landmark extraction, enabling solutions that require only a camera and a computing unit. Neural network models consistently achieve high performance, often exceeding 90% accuracy in both static and dynamic sign classification, confirming the technological feasibility of automated vocabulary recognition. This work presents an integrative systematic review of SRS research motivated by sign language teaching and learning applications, synthesizing 55 articles selected from an initial pool of 161 records identified in the Scopus database between 2020 and 2025. Unlike previous reviews that focused on isolated components of the SRS pipeline or specific sign languages, this review covers the complete workflow—from data capture modality and feature extraction to classification architecture and technological deployment. Its principal contributions are as follows: a structured taxonomy of SRS components and their relative prevalence; a consolidated performance evaluation of static and dynamic sign recognition models; the characterization of a critical deployment gap, with only 11.2% of identified studies reporting integration into user-accessible technologies; and the identification of a geographic concentration of research that leaves the sign languages of Latin American and African communities largely underrepresented. Nevertheless, several persistent challenges remain open. The generalization problem is the most critical: the majority of SRS are trained and evaluated on small, controlled datasets captured under uniform conditions, making robustness to real-world variability in lighting, background, signer appearance, and camera angle difficult to assess. Vocabulary scalability also remains unresolved, as performance degrades substantially as the number of recognizable signs increases, particularly for dynamic signs involving motion and co-articulation. A further structural problem is the absence of standardized evaluation protocols: the reviewed studies report accuracy almost exclusively, omitting precision, recall, F1-score, and inference time, which are essential for assessing deployment readiness and for enabling meaningful cross-study comparisons. Bridging the gap between experimental performance and real-world accessibility requires a sequenced set of engineering priorities. As a foundational step, the field requires large-scale, signer-independent datasets for underrepresented sign languages, collected under diverse environmental conditions and released publicly to enable reproducible benchmarking. Building on this, standardized multi-metric evaluation protocols—reporting precision, recall, F1-score, and inference latency alongside accuracy—should be adopted as a reporting norm, enabling meaningful cross-study comparison and identifying which architectures are genuinely deployment-ready. With these foundations in place, engineering efforts should prioritize model compression and efficiency techniques (pruning, quantization, knowledge distillation) to translate high-performing but computationally expensive architectures, such as Transformers, into forms suitable for mobile and edge deployment. Finally, deployment efforts should be validated through usability studies conducted directly with deaf community members, ensuring that engineering priorities are driven by end-user needs rather than experimental convenience. We propose this sequence—data and evaluation standardization, efficiency-oriented model engineering, and stakeholder-driven deployment validation—as a concrete roadmap for future work in this field.

## Figures and Tables

**Table 1:** Inclusion and exclusion criteria applied in the literature search.

Dimension	Inclusion Criteria	Exclusion Criteria
Database	Scopus	Any source outside Scopus
Publication period	2020–2025	Published before 2020 or after 2025
Language	English, Spanish	Any other language
Access type	Open Access only	Articles behind paywalls
Thematic field	Engineering, Computer Science	Medicine, social sciences, humanities, and other fields
Document type	Journal articles	Conference papers, book chapters, theses

**Table 2:** Studies reported in the literature that involve the development of a sign recognition system.

Sensor	Predictive Model	Result	Sign Language	Reference
Camera	DenseNet121, ResNet152, MobileNetV2, Xception, InceptionV3, NASNetLarge, VGG19, VGG16	A bidirectional automatic translation framework for Arabic Sign Language (ArSL) was designed and implemented using deep learning models and a fuzzy string-matching scoring method. The prototype successfully translated sign images into text and vice versa, achieving up to 98.65% accuracy with VGG16.	Arabic SL	[12]
Camera	LSTM	LSTM models together with MediaPipe were used to recognize ArSL gestures. The model achieved accuracies above 85% for individual volunteers and 83% with combined data.	Arabic SL	[13]
Camera	CNN	A CNN with squeeze-and-excitation blocks and a mobile app were developed. The model reached 99.86% accuracy on the KU-BdSL dataset; SHAP analysis confirmed reliance on hand-related visual cues.	Bangladeshi SL	[14]
Camera	Detectron2, EfficientDet-D0, YOLOv7	An automatic Bangla SL detection system using deep learning and a Jetson Nano was developed. Detectron2 achieved mAP@ of 94.915; YOLOv7 Tiny enabled real-time deployment.	Bangladeshi SL	[15]
Camera	Faster RCNN, YOLOv5, MobileNetV2 (features), LSTM (classification)	An automated BISINDO recognition system robust to backgrounds and computationally efficient. Sentence accuracy: 49.29%; SacreBLEU: 67.77%, outperforming baselines.	Indonesian SL	[16]
Camera	SVM	Dynamic Japanese SL alphabet recognition using feature extraction/selection plus SVM. Achieved 97.20% and 98.40% on two datasets.	Japanese SL	[17]
Data glove	PLD-CNNs	Wearable gloves + deep CNNs for sentence-level Thai SL. Excellent precision, recall, accuracy, and F1 reported.	Thai SL	[18]
Camera	CNN	New CNN architecture achieved 99.7%, setting a new benchmark.	Arabic SL	[19]
Camera	InceptionV3 + LSTM	Lightweight translation framework (LiST) integrating hand gestures, facial expressions, and orientation from Indian SL videos. Translation accuracy 91.2%, prediction 95.9%.	Indian SL	[20]
Camera	CNN + RNN	Dataset of 20 Arabic words; combined CNN-RNN achieved 98% on proposed data and 93.4%/98.8% top-1/top-5 on UCF-101.	Arabic SL	[21]
Camera	SSD + VGG16	Object detection model adapted for AASL. Recognition accuracy 98%; 25% efficiency improvement in real time.	Arabic SL	[22]
Camera	GRU, LSTM, Transformers	Comparative study; LSTM outperformed others with 85.4% average accuracy using augmentation.	American SL	[23]
Camera	YOLOv8	ASL alphabet recognition with MediaPipe + YOLOv8; accuracy 98%, recall 98%, F1 99%.	American SL	[24]
Radar	CNN	Radar-based recognition; 92.31% with unsegmented spectrograms.	–	[25]
Camera	ResNet-50 + VGG-19	Ensemble CNN for 42 Kazakh signs; 95.7% accuracy.	Kazakh SL	[26]
Camera	Transformers	AI-based translator; proof of concept for emergency calls with >200 phrases.	American SL	[27]
Camera	LSTM	Webcam + LSTM for real-time action detection; 99.35% accuracy.	American SL	[28]
Camera	CNN	HVCNNM achieved 99.23% and 99.00% on MUD and ASLAD.	American SL	[29]
Camera	MobileNetV2	New architecture for ASL and ISL alphabets; 98.77%.	American and Indian SL	[30]
Depth camera	CNN	ArSL alphabet recognition; 97.07%.	Arabic SL	[9]
Camera	CNN, LSTM, GRU	Two DL methods; 89.07% (CNN) and 94.3% (LSTM).	American SL	[31]
Camera	CNN	Custom ISL dataset; loss 0.0178, accuracy 99%.	Indian SL	[32]
Camera	RNN	Mexican SL with hand/facial tracking; 0.93 offline, superior online.	Mexican SL	[10]
Camera	LRCN, ConvLSTM	ISL words, only hand gestures, 96.4% accuracy on LSTM model	Indian SL	[33]
Camera	LSTM	Portuguese SL as a service; 80–95.6% accuracy; good usability and semantic correlation with LLM.	Portuguese SL	[34]
Camera	CNN	AI-based system; 97.3% for Kazakh alphabet.	Kazakh SL	[35]

**Table 3:** Most frequently referenced sign language datasets in the reviewed literature (2020–2025).

Dataset	Sign Language	Modality	Sign Type	# Classes	# Samples	References
WLASL	American SL	RGB video	Dynamic	2000	21,083	[58]
AUTSL	Turkish SL	RGB-D video	Dynamic	226	38,336	[3]
LSA64	Argentinian SL	RGB video	Static/Dynamic	64	3200	[41]
KU-BdSL	Bangladeshi SL	RGB image	Static	36	—	[14]
ArSL	Arabic SL	RGB image	Static	32	—	[9,19]

**Table 4:** Performance of models for dynamic sign recognition.

References	Accuracy	# Signs
[13]	0.85	50
[23]	0.85	23
[44]	0.98	20
[10]	0.93	20
[34]	0.95	50
[33]	0.97	8
[17]	0.98	46
[21]	0.92	20

**Table 5:** Performance of models for static sign recognition.

References	Accuracy	# Signs
[12]	0.99	14
[26]	0.98	42
[30]	0.99	26
[9]	0.97	28
[42]	0.99	21
[8]	0.99	36
[63]	0.95	37
[64]	0.98	36

## Data Availability

The original contributions presented in this study are included in the article. Further inquiries can be directed to the corresponding author.
